# Automated volumetric assessment of pituitary adenoma

**DOI:** 10.1007/s12020-023-03529-x

**Published:** 2023-09-25

**Authors:** Raffaele Da Mutten, Olivier Zanier, Olga Ciobanu-Caraus, Stefanos Voglis, Michael Hugelshofer, Athina Pangalu, Luca Regli, Carlo Serra, Victor E. Staartjes

**Affiliations:** 1 Machine Intelligence in Clinical Neuroscience (MICN) Laboratory, Department of Neurosurgery, Clinical Neuroscience Center, University Hospital Zurich, University of Zurich, Zurich, Switzerland; 2 Department of Neurosurgery, University Hospital Zurich, Frauenklinikstrasse 10, 8091 Zurich, Switzerland; 3 Department of Neuroradiology, University Hospital Zurich, Frauenklinikstrasse 10, 8091 Zurich, Switzerland

**Keywords:** Pituitary, Extent of resection, Machine learning, Deep learning, Endocrinology, Neurosurgery

## Abstract

**Purpose:**

Assessment of pituitary adenoma (PA) volume and extent of resection (EOR) through manual segmentation is time-consuming and likely suffers from poor interrater agreement, especially postoperatively. Automated tumor segmentation and volumetry by use of deep learning techniques may provide more objective and quick volumetry.

**Methods:**

We developed an automated volumetry pipeline for pituitary adenoma. Preoperative and three-month postoperative T1-weighted, contrast-enhanced magnetic resonance imaging (MRI) with manual segmentations were used for model training. After adequate preprocessing, an ensemble of convolutional neural networks (CNNs) was trained and validated for preoperative and postoperative automated segmentation of tumor tissue. Generalization was evaluated on a separate holdout set.

**Results:**

In total, 193 image sets were used for training and 20 were held out for validation. At validation using the holdout set, our models (preoperative / postoperative) demonstrated a median Dice score of 0.71 (0.27) / 0 (0), a mean Jaccard score of 0.53 ± 0.21/0.030 ± 0.085 and a mean 95^th^ percentile Hausdorff distance of 3.89 ± 1.96./12.199 ± 6.684. Pearson’s correlation coefficient for volume correlation was 0.85 / 0.22 and −0.14 for extent of resection. Gross total resection was detected with a sensitivity of 66.67% and specificity of 36.36%.

**Conclusions:**

Our volumetry pipeline demonstrated its ability to accurately segment pituitary adenomas. This is highly valuable for lesion detection and evaluation of progression of pituitary incidentalomas. Postoperatively, however, objective and precise detection of residual tumor remains less successful. Larger datasets, more diverse data, and more elaborate modeling could potentially improve performance.

## Introduction

Pituitary adenomas (PA) are a frequent type of intracranial tumor [1]. Endonasal transsphenoidal surgery has established itself as the best option for its treatment in most cases [2]. Its outcome varies greatly with different factors like tumor morphology and the surgeon caseload [3–5]. Treatment is indicated in case of functioning PA other than prolactinomas, in case of symptomatic PA or in case of relevant volumetric progression [6]. If surgery is performed, assessment of residual tumor is relevant in order to determine the extent of resection (EOR) [7], though manual segmentation of tumor volumes is likely highly dependent on the rater, especially postoperatively [8–10]. Automated analysis of pre- and postoperative imaging could consequently have the potential to provide more objective and precise volumetry.

Semantic image segmentation is a classic machine learning application [11, 12], not only due to the fact that manual segmentation requires considerable amounts of expert time [13, 14]. Convolutional neural networks (CNNs)—and specifically U-Nets—have recently been applied successfully for biomedical image segmentation due to their throughput speed and overall good performance in this task [15].

To the best of the authors’ knowledge, no automated approaches to segment PA pre- and postoperatively for volumetry and resection assessment exist. We hypothesize that a CNN can generate segmentations of PA faster and more objective while maintaining quality of segmentation.

## Methods

### Data and preprocessing

Patients undergoing transsphenoidal surgery for PA at University Hospital Zurich in the period of October 2012 to May 2021 with available preoperative and 3-month postoperative 3-Tesla magnetic resonance imaging (MRI) were included. After identifying the closest T1-weighted contrast-enhanced MRI scan prior to surgery and a three month follow up those were assigned a Study ID and exported. In order to account for different manufacturers and acquisition protocols at referring hospitals, the images were converted to NIfTI format [16], reshaped to 256 × 256 × 256 voxels, voxel size normalized to 1.0 × 1.0 × 1.0, and images were reoriented using a right-anterior-superior affine matrix. Tumor tissue was subsequently manually labeled for training and residual volume assessment. After creating a holdout set of 20 patients for assessment of model generalization, the remaining 386 studies (two per patient) had its pixel intensities normalized for each study individually, and were then sliced in the coronal plane [17].

### Model Development

We used a 2D-U-Net as model architecture, with a binary cross entropy loss function, Adam optimizer, and a sigmoid activation function [15]. It was built using the following platforms: Python 3.9.0 [18], Keras 2.5.0 [19], SimpleITK [20–22] and nibabel [23]. Training was carried out on a Nvidia RTX 3090 graphical processing unit. Separate preoperative and postoperative models were trained in five-fold cross validation.

**Fig. 1:**
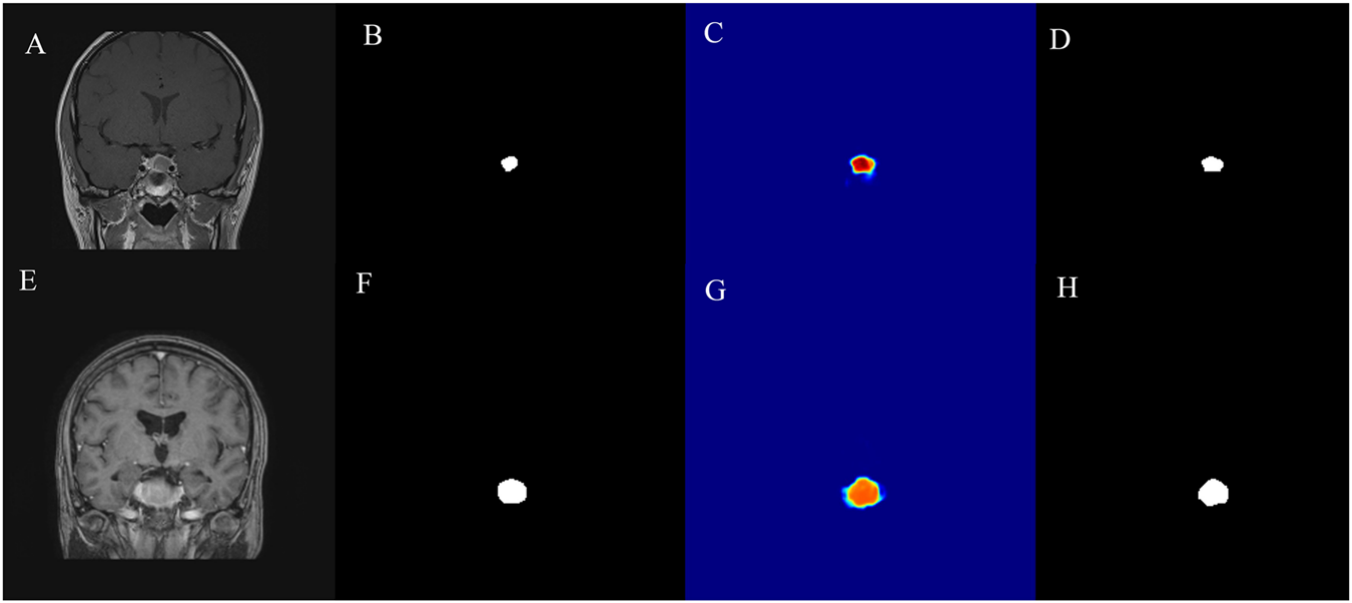
Examples of images and predictions. A/E preoperative input image in coronal orientation, B/F ground truth, C/G unthresholded predictions, D/H thresholded predictions

The five resulting models were subsequently used to create ensemble segmentations by averaging their respective predictions. To binarize the predicted probabilities ranging from zero to one, a threshold of 0.6 for preoperative and 0.44 for postoperative scans was used as illustrated in Fig. 1. For the postoperative predictions, the following automated postprocessing steps were implemented: Coherent volumes smaller than fifty pixels were removed, holes within the segmentation were filled and a dilate function was used to smoothen the corners which is closer to natural tumor growth.

For the postoperative models, we additionally implemented transfer learning by initializing the postoperative models with the parameters of the fully trained preoperative models, and image augmentation was performed with a sampling ratio of 1/255 and rotation of between 0 and 90 degrees as well as zoom of 0% to 30%.

### Evaluation

Manual and automated segmentations were compared using the Dice score, Jaccard score and the 95th percentile of the Hausdorff distance [24–27]. Dice and Jaccard evaluate similarity and overlap and range from zero—indicating no overlap—to one for perfect congruence. The Hausdorff distance analyzes the distances between two sets of points made up from the edges of two segmentations. Smaller values thus represent better performance. We opted for the 95th percentile instead of maximal Hausdorff distance to decrease the importance of outliers. Volumes were calculated in mm^3^ from the segmented masks, and their correlation with the manually segmented volumes was assessed using Pearson’s product-moment correlation. Automated and manual EOR were similarly correlated. Finally, we assessed the model’s performance in detecting gross total resection (GTR, defined as an EOR of 100%) using a confusion matrix.

## Results

### Cohort

In total, 213 patients were included retrospectively, of which 193 were applied for development of the model. Validation was performed in 20 held-out patients. Summary demographics and radiological information are displayed in Table 1.

**Table 1:** Summary of the patient,tumor and radiological characteristics

Variable		Cohort
	Test / Train Set	Holdout Set
Baseline		
Number of Patients	193	20
Age years, mean ± SD	55 ± 19	52 ± 20
Male gender, n (%)	107 (55)	13 (65)
Biochemical activity		
NFPA, n (%)	113 (58.55)	15 (0.75)
GH, n (%)	45 (23.32)	4 (20)
PRL, n (%)	19 (9.84)	0 (0)
ACTH, n (%)	8 (4.14)	0 (0)
Others, n (%)	8 (4.14)	1 (5)
ZPS score		
ZPS I, n (%)	61 (31.61)	0 (0)
ZPS II, n (%)	82 (42.49)	9 (45)
ZPS III, n (%)	31 (16.06)	8 (40)
ZPS IV, n (%)	11 (5.70)	1 (5)
Score missing, n (%)	8 (4.14)	2 (10)
Knosp score		
Knosp 0, n (%)	44 (22.80)	1 (5)
Knosp 1, n (%)	43 (22.28)	7 (0.35)
Knosp 2, n (%)	47 (24.35)	6 (0.3)
Knosp 3, n (%)	35 (18.13)	4 (20)
Knosp 4, n (%)	16 (8.29)	1 (5)
Score missing, n (%)	8 (4.14)	1 (5)
EOR		
EOR mean ± SD	92.84 ± 29.21	97.77 ± 3.60
EOR median (IQR)	100.00 (4.18)	99.57 (3.04)
Tumor volume		
Preoperative, mean ± SD (ml)	7.42 ± 9.93	9.12 ± 8.86
Preoperative, median (IQR) (ml)	4.79 (7.1)	6.37 (7.75)
Postoperative, mean ± SD (ml)	0.35 ± 1.61	0.67 ± 0.84
Postoperative, median (IQR) (ml)	0 (0.05)	0.06 (1.36)
MRI data		
Number of scans	386	40
Vendor		
Siemens	171	0
Philips Healthcare	144	36
GE Medical Systems	71	4
Acquisition orientation		
2D	317	31
3D	69	9
Field strength		
1 T	1	1
1.5 T	45	6
3 T	340	33
Voxel dimensions		
Pixel Spacing, mean ± SD (mm)	0.47 ± 0.14	0.51 ± 0.14
Pixel Spacing, median (IQR) (mm)	0.45 (0.19)	0.47 (0.09)
Slice Thickness, mean ± SD (mm)	1.98 ± 0.53	2.02 ± 0.67
Slice Thickness, median (IQR) (mm)	2 (0)	2 (0)

*SD* standard deviation, *IQR* interquartile range, *NFPA* non functioning pituitary adenoma, *GH* growth hormone, *ACTH* adrenocorticotropic hormone, *PRL* prolactin, *GTR* gross total resection, *T* Tesla

### Preoperative segmentation performance

**Fig. 2:**
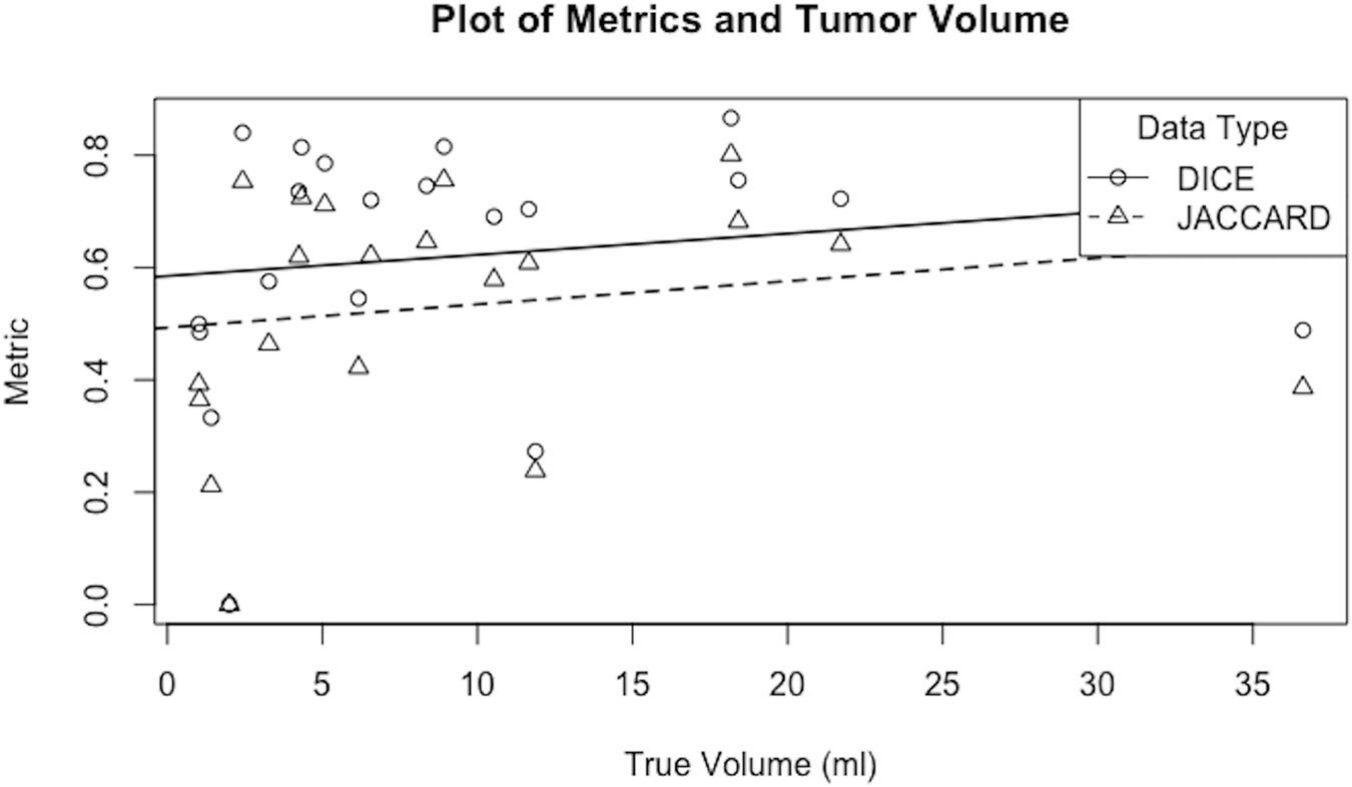
Metric performance and manually segmented tumor volume of the holdout set with a linear regression

Segmentation performance is summarized in Table 2. In terms or preoperative tumor segmentation, our ensemble model achieved a mean Dice score of 0.62 ± 0.22, with automatically rated volumes correlating well with manually segmented volumes (r = 0.85). Figure 2 shows metric performance and tumor volume with a linear regression. Exact Jonckheere-Terpstra-Test for a trend did not reach significance (JT, *p* value for Dice score: 110, 0.1757; JT, *p* value for Jaccard score: 114, 0.1166) [28].

**Table 2:** Segmentation performance of the fully trained preoperative and postoperative models

Metric	Preoperative U-Net		Postoperative U-Net	
Performance type	Resampled training	Holdout set	Resampled training	Holdout set
Dice				
Mean ± SD	0.50 ± 0.05	0.62 ± 0.22	0 ± 0	0.046 ± 0.125
Median (IQR)	0.49 (0.05)	0.71 (0.27)	0 (0)	0 (0)
Jaccard				
Mean ± SD	0.43 ± 0.05	0.53 ± 0.21	0 ± 0	0.030 ± 0.085
Median (IQR)	0.43 (0.05)	0.61 (0.30)	0 (0)	0 (0)
95th percentile Hausdorff distance				
Mean ± SD	6.55 ± 0.70.	3.89 ± 1.96	20.810 ± 14.604	12.199 ± 6.684
Median (IQR)	4.50 (1.13)	3.39 (1.66)	15.769 (12.152)	0 (6.655)

Resampled training performance as well as generalization towards the held-out data is reported

*SD* standard deviation, *IQR* interquartile range

### Postoperative segmentation performance

For postoperative segmentations, a mean Dice score of 0.046 ± 0.125 was observed. The correlation of manually segmented tumors and predicted tumor masks correlated less satisfactory than in preoperative scans (r = 0.22). Introduction of transfer learning techniques and image augmentation did not improve performance (Table 3).

**Table 3:** Image augmentation and transfer learning based on the preoperative models were applied as an attempt to improve postoperative segmentation performance

Metric	Postoperative U-Net + Augmentation	Postoperative U-Net + Transfer Learning	Postoperative U-Net + Augmentation + Transfer Learning
Performance Type	Holdout Set	Holdout Set	Holdout Set
Dice			
Mean ± SD	0 ± 0	0.040 ± 0.087	0.013 ± 0.035
Median (IQR)	0 (0)	0 (0.007)	0 (0.007)
Jaccard			
Mean ± SD	0 ± 0	0.026 ± 0.062	0.008 ± 0.022
Median (IQR)	0 (0)	0 (0.004)	0 (0.004)
95th percentile Hausdorff distance			
Mean ± SD	NaN	15.051 ± 8.131	14.876 ± 5.021
Median (IQR)	NaN	13.045 (7.836)	15.953 (8.050)

Generalization towards the held-out data is reported

*SD* standard deviation, *IQR* interquartile range

### Resection assessment performance

Table 4 summarizes performance in terms of resection assessment. Our model’s predictions generated EOR values that only poorly correlated with manual segmentations (r = −0.14). Automatically detected EOR differed from the ground truth manual segmentation by 18.65% ± 31.10% on average. GTR was detected with an accuracy of 50.00%, sensitivity of 66.67% and specificity of 36.36%.

**Table 4:** Volumetric performance of the automated resection assessment pipeline

Measurement	U-Net Performance vs. Manual Segmentation
Preoperative Volume [mm^3^]	
Correlation (Pearson)	0.85
Postoperative Volume [mm^3^]	
Correlation (Pearson)	0.22
EOR [%]	
Correlation (Pearson)	−0.14
Difference in EOR [Median (IQR)]	3.31% (17.75%)
Difference in EOR [Mean ± SD]	18.65% ± 31.10%
GTR	
Accuracy	50.00%
Sensitivity	66.67%
Specificity	36.36%
PPV	46.15%
NPV	57.14%

*EOR* extent of resection, *GTR* gross total resection, *PPV* positive predictive value, *NPV* negative predictive value

*SD* standard deviation, *IQR* interquartile range

## Discussion

We have developed and validated an automated PA segmentation pipeline based on deep learning. We demonstrate that our approach performs favorably when it comes to segmentation and volumetric assessment of preoperative images. Generating objective and precise postoperative segmentations of residual tumor remains a challenge, even with the application of advanced machine learning techniques.

As neuroimaging has become much more frequent, also detection of incidentalomas is prone to increase since especially nonfunctioning incidentaloma of the pituitary are highly prevalent (1.4–27% in autopsy and 3.7–37% in imaging) [29]. Hence automated segmentation of incidental PA would tackle a frequent issue. Incorporating this into a diagnostic software to detect suspicious lesions of the pituitary gland would be valuable for clinical routine. To address this, we developed a fully automated graphical user interface that tackles this issue (https://micnlab.com/download-the-zurich-neurosurgical-toolkit/).

Furthermore, automated, objective volumetric measurement for assessing progression of incidentalomas, especially microadenomas, yields a clinical benefit since volumetric progression is crucial for surgical indication [30]. Last, prognostic scores like the Zurich pituitary or Knosp score could be automatically implemented, ultimately giving the clinician further information and saving his time. These three options combined have the potential to standardize and speed up the clinical workflow of PA.

When indicated, the transsphenoidal approach is very effective in most cases of PA with comparatively low surgical morbidity and mortality [3]. When evaluating surgical oncology results—not only in individual patients, but also when comparing cohorts, surgeons, and departments and for research purposes—volumetric assessment of residual adenoma volume is of paramount importance. However, segmenting tumors on each slice accurately is time-consuming and often not possible in daily clinical practice. Furthermore, as in other tumor segmentation tasks such as gliomas, the interrater agreement likely is low, especially for postoperative residual tumor tissue [10, 31]. In this light, it must be considered that even morphological grading at the preoperative stage using the Hardy and Knosp classification already suffers from relatively low interrater agreement [8, 9].

Volumetric rating of post-resection sellar scans thus presents a particular challenge when it comes to objectivity. Automated semantic segmentation – the machine learning task that deals with detecting and outlining structures on images—could prove a viable option to improve the speed and objectivity with which volumes are assessed pre- and postoperatively.

To some extent, the poor performance on postoperative images is to be expected: In the end, supervised learning techniques can only ever be as good as the “ground truth” data they are trained on, and with disagreements in labeling of small or debatable residual tumor, this has certainly been the case in this study. Even in the much more intensely studied task of glioma and glioblastoma segmentation, which has been fueled by the yearly international BraTS challenges [32], performance overall appears mediocre and demonstrates that – especially for low grade glioma—it appears to be difficult to generate any improvements in segmentation compared to human raters, apart from the increased speed and objective nature with which automated segmentations can be produced. Even in BraTS 2014/2015, where postoperative images were also segmented, the ground truth labels for postoperative images eventually had to be generated by learning algorithms.

Our ensemble method is—to the best of the authors’ knowledge—the only attempt at automatically segmenting post-transsphenoidal surgery scans. The deep learning approach was able to accurately outline the tumors preoperatively, but struggled with small residual tumors. Certainly, this can be explained at least partially by the computationally necessary downsampling of the images, resulting at times in voxels that appear almost equally as large as the residual tumor itself. Also, pituitary adenomas do not appear constantly with the same relative intensity making them hard to identify at times [33].

### Limitations

Although we included scans from many institutions and all major scanner manufacturers, our study remains single-center and external validation would be necessary before any kind of clinical application. Furthermore, we applied 2D segmentation—which has demonstrated reliable results previously for other similar applications—although 3D segmentation potentially could further increase performance. A larger dataset of subtotally resected pituitary adenomas would most likely also allow improvements, since most postoperative images showed no tumor for the model to learn to recognize. A more heterogeneous dataset as we did allows for better generalization and reduces the risk of overfitting to a particular manufacturer or hospital protocol. On the other hand, a reduction in performance is to be expected with this approach.

## Conclusion

Our volumetry pipeline demonstrated its ability to accurately and automatically segment pituitary adenoma. This is highly valuable for lesion detection and evaluation progression of pituitary incidentalomas. Postoperatively, however, objective and precise detection of residual tumor remains less successful. Larger datasets, more diverse data, and more elaborate modeling could potentially improve performance. Yet, focusing on use cases for preoperative segmentations seems more promising.

## Data Availability

The data in support of our findings can be obtained upon reasonable request from the corresponding author. The models can be downloaded and applied using our graphical user interface, available at “https://micnlab.com/download-the-zurich-neurosurgical-toolkit/”.
